# Ultra-low detection limit chemoresistive NO_2_ gas sensor using single transferred MoS_2_ flake: an advanced nanofabrication†

**DOI:** 10.1039/d2ra06228c

**Published:** 2022-11-22

**Authors:** Hoang Si Hong, Tran Vinh Hoang, Nguyen Thanh Huong, Nguyen Hoang Nam, Dao Duc Thinh, Nguyen Thi Hue, Nguyen Duc Thuan

**Affiliations:** School of Electrical and Electronic Engineering, Hanoi University of Science and Technology (HUST) No. 1 Dai Co Viet Road Hanoi Vietnam hong.hoangsy@hust.edu.vn +84-43-869-6211; School of Chemical Engineering, Hanoi University of Science and Technology (HUST) No. 1 Dai Co Viet Road Hanoi Vietnam

## Abstract

In this work, a method of fabricating a NO_2_ nano-sensor working at room temperature with a low detectable concentration limit is proposed. A 2D-MoS_2_ flake is isolated by transferring a single MoS_2_ flake to SiO_2_/Si substrate, followed by applying an advanced e-beam lithography (EBL) to form a metal contact with Au/Cr electrodes. The resulting chemoresistive nano-sensor using a single MoS_2_ flake was applied to detect a very low concentration of NO_2_ at the part-per-billion (ppb) level. This result is obtained due to the ability to create microscopic nano-sized MoS_2_ gaps using e-beam lithography (300 nm–400 nm). Experimental results also show that the sensor can capture changes in concentration and send the information out extremely quickly. The response and recovery time of the sensor also reached the lowest point of 50 and 75 ms, outperforming other sensors with a similar concentration working range.

## Abstract 2

In this work, a method of fabricating a NO_2_ nano-sensor working at room temperature with a low detectable concentration limit is proposed.

## Graphical abstract

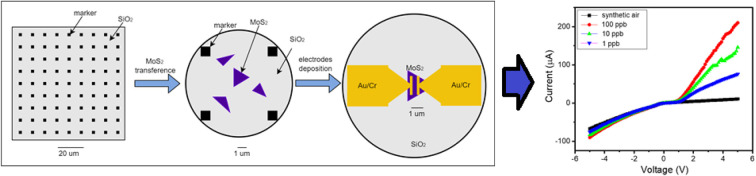

## 1. Introduction

The amount of poisonous gases emitted into the environment has significantly increased due to industrial development as well as improvements in quality of life and transit accessibility, causing substantial harm to the ecosystem and the general public, making it necessary to monitor the air quality in metropolitan areas.^1,2^ Among the aforementioned air contaminants, NO_2_ is one of the most deadly as at concentrations over 1 ppm, it causes or aggravates respiratory disorders including emphysema and bronchitis as well as severe damage to lung tissues in humans.^3^ Therefore, research on gas sensors is helpful for the NO_2_ emission monitoring and controlling process and is a potential research direction where a large number of scientists are currently interested.

Two-dimensional (2D) materials have been applied in various research applications and industries because of their potential in chemical gas sensing, electronics, optoelectronics, optics, and energy generation.^4–7^ In previous works, the resistive NO_2_ gas sensor was fabricated using 2D materials (molybdenum disulfide MoS_2_/graphene hybrid), which showed high sensitivity, good selectivity, and a low detection limit.^8^ However, the concentration limit of that type of sensor is still relatively high and is not suitable for monitoring NO_2_ in the surrounding environment. The nano-sensor deployed by the combination of two-dimensional (2D) materials of atomic-layer thickness and the advantages of nanofabrication is an urgent research direction because of excellent outcomes in the chemical gas sensing field. However, the biggest challenge in this research direction is how we can make sensor devices at the nanoscale to quickly trap these changes in 2D materials, *i.e.*, what is the optimal design and what are the proper materials. This has been the motivation for us to conduct experiments to fabricate a highly sensitive nanoscale NO_2_ gas sensor using advanced materials.

MoS_2_ has recently been suggested as a promising material for NO_2_ gas sensing^9–12^ because, when combined with graphene to create composite or hybrid materials, it increases sensitivity due to synergistic effects and eliminates the issue of high recovery time of graphene-only NO_2_ sensor.^13^ Moreover, the challenge in developing chemoresistive sensors is the distance between electrodes, which is limited by e-beam lithography technology. The short distance between electrodes and the sensing area at truly nanoscale give such advantages as fast response and low detection limit.^14^ In this work, we transferred a single MoS_2_ flake to SiO_2_/Si and applied advanced e-beam lithography to make chemoresistive nanosensors to detect a very low concentration of NO_2_ gas.

## 2. Experimental section

### 2.1 Transferred MoS_2_ and fabrication of chemoresistive nanosensor

**Fig. 1:**
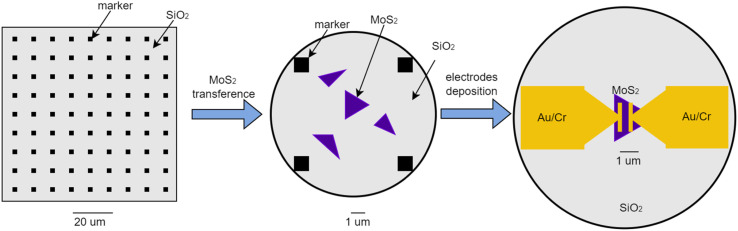
The illustration of the chemoresistive NO_2_ nanosensor fabrication process.

As previously mentioned,^8^ MoS_2_ was deposited together with the CVD-based synthesis of graphene utilizing copper foil and transferred to SiO_2_/Si. To fabricate a chemoresistive NO_2_ gas sensor, two steps of e-beam lithography were applied. In the first step, the array of Au/Cr marker was fabricated on SiO_2_/Si using 100 nm thick PMMA as e-beam resist. Then, the single MoS_2_ flake was transferred to SiO_2_/Si (with Au/Cr marker on top) using 3M Scotch tape. The intermediate SEM observation step is to find the MoS_2_ flake on SiO_2_/Si after the transferring process and identify the MoS_2_ flake position using Au/Cr marker. After identifying the MoS_2_ flake location on the array Au/Cr marker with high precision, the second e-beam step was performed on PMMA (100 nm)/MoS_2_/SiO_2_/Si to make metal contact by the lift-off process. Then, two Au/Cr electrodes were deposited on MoS_2_/SiO_2_/Si by e-beam evaporation to make the final chemoresistive nano-sensor device. The illustration of chemoresistive NO_2_ nanosensor fabrication process is shown in Fig. 1.

### 2.2 Evaluation methods

The morphology and nanofabrication of the MoS_2_/SiO_2_/Si sample were determined using high-resolution scanning electron microscopy (HR-SEM; SU 8010, Hitachi) and high-resolution e-beam lithography (e-Raith 150). High-resolution transmission electron microscopy (HR-TEM; JEM-2100F, JEOL) was used to gauge the atomic structure and thickness of transferred MoS_2_. Raman spectroscopic measurements were conducted in WITec Raman imaging with a 532 nm laser source to identify MoS_2_ quality before/after the transferring process. The NO_2_ gas sensor was installed inside a sealed chamber that was attached to NO_2_ gas cylinders with various concentrations. The *I*–*V* characteristics and resistance of the sensor were recorded using a semiconductor probe station (Keithley SCS-4200). The detailed measurement setup for the NO_2_ gas sensor was described in previous work.^8^

## 3. Results and discussion

**Fig. 2:**
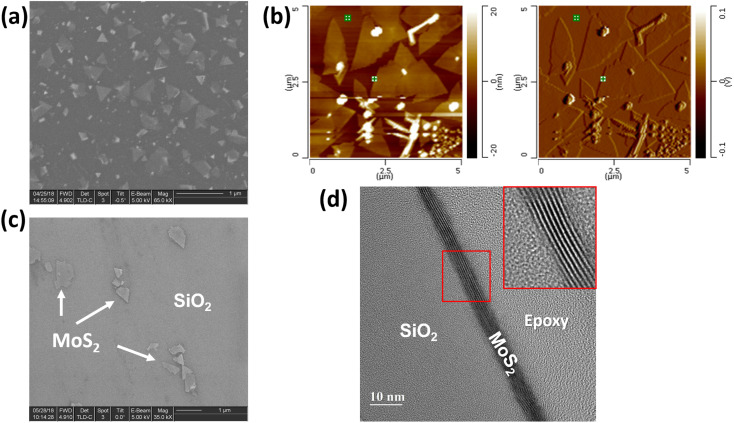
MoS_2_ flakes deposited on graphene: (a) SEM image, (b) AFM analysis, (c) SEM images of isolated MoS_2_ flake on SiO_2_/Si after the transferring process and (d) cross-sectional view of HR-TEM analysis of transferred MoS_2_ on SiO_2_/Si (with the help of focused-ion-beam (FIB) to prepare sample specimen).

As-grown MoS_2_–graphene hybrid and a single MoS_2_ flake on SiO_2_/Si after the transfer process are depicted in SEM images in Fig. 2(a). It is obvious that the MoS_2_ with 2D crystallite was grown on graphene and was triangle-shaped. The triangle-like MoS_2_ flakes are with average sizes of around 1–2 μm. The MoS_2_ flakes were grown in a large-scale area on graphene with uniform thickness as shown in AFM images in Fig. 2(b). In Fig. 2(c), the transferred MoS_2_ seems to induce defects during the transfer process by breaking into smaller flakes compared with the original MoS_2_ flake as-deposited on graphene (see AFM image in Fig. 2(b)). In order to measure the thickness of MoS_2_ flakes on graphene, the AFM with tapping mode was carried out. Results in MoS_2_ flake average thickness is around 5 nm, according to 10 layers of MoS_2_, consistent with the report in ref. 8. Fig. 2(d) shows the cross-sectional view of HR-TEM of transferred MoS_2_ on SiO_2_/Si substrate, which indicates 8 layers of MoS_2_.

**Fig. 3:**
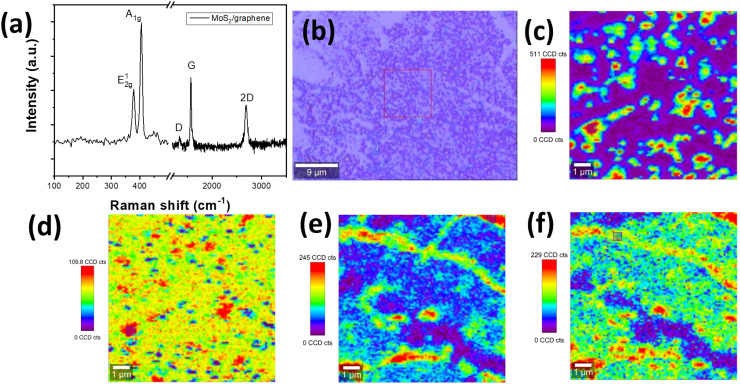
(a) Raman spectra of MoS_2_–graphene on SiO_2_/Si and (b) optical image with selected area for Raman mapping, (c) Raman mapping for A_1g_ mode, (d) Raman mapping for E_g_ mode of MoS_2_, Raman mapping for (e) G peak and (f) 2D peak of graphene.

Fig. 3 shows the Raman spectra of a few layers of MoS_2_ growth on graphene and their Raman mapping. In Fig. 3(a), two Raman peaks were assigned to MoS_2_ material (E_g_ at ∼378.5 cm^−1^ and A_1g_ at ∼405.8 cm^−1^).^8^ The E_g_ modes are doubly degenerate in-plane vibrational modes and A_1g_ is an out-of-plane vibration mode, which agrees well with previous observations in β-phase bulk MoS_2_.^15^ In comparison with the Raman spectrum of transferred pure MoS_2_ flakes (transferring from MoS_2_/graphene/SiO_2_/Si sample) on SiO_2_/Si substrate, both peak frequencies of β-phase (E_g_ and A_1g_) of MoS_2_–graphene shift to the higher wavenumber region. The Raman peaks of transferred MoS_2_ flakes on SiO_2_/Si, as shown in Fig. S1,† are at 370.9 and 401.2 cm^−1^ for E_g_ and A_1g_ mode, respectively. The shift in Raman peaks of MoS_2_ material is due to either a strain/stress free state after transferring from graphene or the influence of the thickness of a few-layers MoS_2_ on Raman shift.^15^ The G and 2D bands of graphene were assigned to two minor peaks at 1584.3 cm^−1^ and 2677.2 cm^−1^, respectively. The in-plane vibration of sp^2^-carbon atoms was attributed to the G band, while defects in graphene were associated with the D band's breathing modes of six-atom rings. The D band, which is at around 1350 cm^−1^ indicating defects in graphene, was not present in the sample after depositing MoS_2_ as in Fig. 3(a). However, the 2D peaks indicating the number of layers of graphene or graphene quality appeared at 2677.2 cm^−1^. The intensities of graphene-related peaks are lower than MoS_2_ peaks because of either the thickness of the few-layer MoS_2_ or the focus point of the laser from Raman. Fig. 3(b) shows an optical image of MoS_2_–graphene and the selected area for Raman mapping. Fig. 3(c and d) are images for Raman mapping for average peak intensity centered at 405.8 cm^−1^ and 378.5 cm^−1^ for A_1g_ and E_g_ mode of MoS_2_ material, respectively. The A_1g_ peak has clear contrast on the area of MoS_2_ flakes in comparison with the E_g_ peak, which indicated the higher intensity of A_1g_ mode in MoS_2_ flakes. Fig. 3(e and f) are images of Raman mapping of G and 2D peaks related to graphene, respectively. In conversion, the mapping of peaks related to graphene has more uniformity in color, except at the graphene grain boundary, which gives higher intensity in Fig. 3(e and f) due to the thicker thickness of folded graphene at the boundary. These Raman mapping images confirm the MoS_2_ as isolated flakes on the base planar graphene layer.

**Fig. 4:**
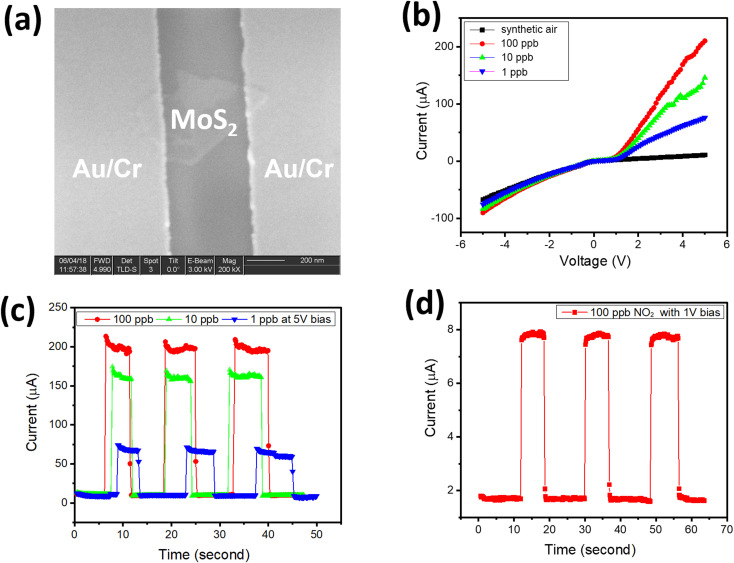
(a) SEM image of the fabricated nanosensor of Au–MoS_2_–Au, (b) *I*–*V* characteristics of sensors with various NO_2_ concentrations, (c) repeatability response of sensor with different NO_2_ concentrations of 1, 10, 100 ppb at 5 V bias (d) transient response to 3 cycles of 100 ppb at 1 V bias.

Fig. 4(a) shows the fabricated chemoresistive sensor using a single transferred MoS_2_ flake. The Au/Cr metal contact on a single MoS_2_ flake is with a distance of 300 nm between two electrodes. The fabricated sensor was tested several times during the day and on different days. Results showed a consistent response with the same testing conditions (NO_2_ gas concentrations and bias voltage). Fig. 4(b–d) shows the sensor responses with a low concentration of NO_2_ gas. The *I*–*V* curves were recorded with synthetic air and different NO_2_ gas concentrations of 1, 10, and 100 ppb as shown in Fig. 4(b). The fabricated sensor showed a clearly different response with various NO_2_ concentrations. Fig. 4(b) indicates the maximum response of the fabricated sensor with 100 ppb NO_2_. *I*–*V* curves in Fig. 4(b) show that the sensor response increased dramatically in the forwarding region when exposed to NO_2_ gas. The non-linear behavior in the *I*–*V* characteristic of Au/MoS_2_/Au sample is due to either space-charge limited current in 2D materials^16^ or oxidized MoS_2_ surface giving the nano-junction with Au electrode. With the same sample structure without MoS_2_ flakes, pure graphene sample (with two Au/Cr electrodes and a distance of 300 nm) shows linear ohmic contact and negligible change when exposed to 100 ppb NO_2_ gas (see Fig. S2 in ESI†). Fig. 4(c) shows the transient response of the fabricated sensor with 5 V bias exposed under 3 repeat cycles of 1, 10, and 100 ppb. The sensor response shows good repeatability and the gas-induced-current increased by two orders of its value when exposed to NO_2_ gas. The response/recovery time of the fabricated sensor is estimated at around 50/75 ms. Fig. 4(d) shows the transient response to 3 cycles of 100 ppb NO_2_ at a lower bias voltage of 1 V. The current increased only 4-fold with 100 ppb NO_2_ at 1 V bias, which is much smaller than with a 5 V bias. The NO_2_ gas generated more current in Au–MoS_2_–Au at a higher bias voltage in comparison with a low bias voltage due to the influence of the external electrical field on electron–hole separation and charge collection at the electrode.^14,16^ The performance of the fabricated NO_2_ gas sensor can be compared with previously developed sensors (Table 1).^17–21^ All of them are sensors that work at NO_2_ concentrations at ppb, so all comparisons are fair. It is evident that the proposed sensor exhibited a low limit of detection and high sensitivity, among others. In addition, the response/recovery time of the proposed sensor also outperforms others by being more than 60 times faster. In Table 1, the sensor response (*S*) at fixed bias voltage is defined as the ratio of current change when the sensor is exposed to NO_2_:

1 *S* = *I*_g_/*I*_a_

where *I*_a_ is the current of the sensor in the presence of synthesized air only, and *I*_g_ is the current in the presence of NO_2_ at given concentrations. The response time is defined as the time required for the sensor to reach 90% of the current change (Δ*I*) when the sensor is exposed to a given concentration of NO_2_. The recovery time is defined as the time needed to recover 90% of the initial baseline after NO_2_ is turned off.

**Table 1:** Comparison of representative NO_2_ gas sensors

Materials	Experimental detected concentration (ppb)	Sensor response (*S* = *I*_g_/*I*_a_)	Response/recovery time	Condition	Ref.
ZnO	25	0.16	—	100 °C UV light	17
ZnO	25	1.65	—	RT UV light	18
Cu_2_O–CuO microflowers-2	10	1.6	75 s/100 s	187 °C 30% RH	19
SnS_2_ nanopetals	5	19.7	179 s/782 s	RT	20
CuO nanoflakes/rGO	50	20.6	31.8 s/60.6 s	RT	21
MoS_2_/SiO_2_	1	5.1	50 ms/75 ms	RT	This work
MoS_2_/SiO_2_	10	12.9	50 ms/75 ms	RT	This work

The sensor response of pure graphene with NO_2_ is shown in Fig. S2(b and c).† Obviously, the Au–MoS_2_–Au showed significantly higher responsivity in comparison with pure graphene (for responsivity of pure graphene, see Fig. S1 in ESI†). The sensor made of Au–MoS_2_–Au has increased responsivity from 4 to 100 with 100 ppb NO_2_ at 1 V and 5 V bias, respectively. Meanwhile, the sensor response of pure graphene is not significant, which is less than 1% in Fig. S2(b and c),† at 1 V and 5 V bias. The selectivity of the fabricated sensor towards NO_2_, in comparison with common toxic gases/moisture such as H_2_, H_2_O, CO, and CO_2_, is shown in Fig. S2(d).† In Fig. S2(d),† examined gases/moisture were measured at 1000 ppb, and the negative sensor response indicates that the current was reduced with H_2_, CO, and CO_2_ gas. Results showed good selectivity of the fabricated sensor towards NO_2_ gas, which is promising for practical applications.

## 4. Conclusions

In this work, MoS_2_ was successfully deposited on graphene by thermal CVD. The single MoS_2_ flake was then transferred successfully to SiO_2_/Si substrate as isolated flakes. Advanced e-beam lithography was applied to fabricate the nanosensor with a single MoS_2_ flake only. The created nanosensor could identify low NO_2_ concentrations between 1 and 100 ppb at room temperature, showing a fast response/recovery of 50/75 ms. Thanks to the recent advantages of nanofabrication in combination with the development of 2D materials, the chemical gas sensor at truly nanoscale showed its advantages with low detection limit and fast response and is promising for detecting NO_2_ at the atomic level in practical applications. In future works, the performance of the fabricated sensor will be considered over a long-term period.

## Conflicts of interest

There are no conflicts to declare.

## Supplementary Material

RA-012-D2RA06228C-s001
