# Supplementary material for: Ultra-low detection limit chemoresistive NO2 gas sensor using single transferred MoS2 flake: an advanced nanofabrication
Source: RSC Adv. 2022 Nov 22;12(51):33403–8. doi: 10.1039/d2ra06228c (PMC9679951; doi:10.1039/d2ra06228c)
Supplement: RA-012-D2RA06228C-s001 [file RA-012-D2RA06228C-s001.pdf]

Electronic Supplementary Material (ESI) for RSC Advances.
This journal is © The Royal Society of Chemistry 2022

Supplementary Information

Ultra-low detection limit chemoresistive NO_2 gas sensor using single transferred MoS_2 flake: an advanced nanofabrication

Hoang Si Hong [a]*, Tran Vinh Hoang [b], Nguyen Thanh Huong [a], Nguyen Hoang Nam [a], Dao Duc Thinh [a], Nguyen Thi Hue [a] and Nguyen Duc Thuan [a]

[a] *School of Electrical and Electronic Engineering, Hanoi University of Science and Technology (HUST), No. 1 Dai Co Viet Road, Hanoi, Vietnam*

[b] *School of Chemical Engineering, Hanoi University of Science and Technology (HUST), No. 1 Dai Co Viet Road, Hanoi, Vietnam*

* Corresponding author. Tel.: +84-43-869-6211

E-mail address: hong.hoangsy@hust.edu.vn *(Hoang Si Hong);*

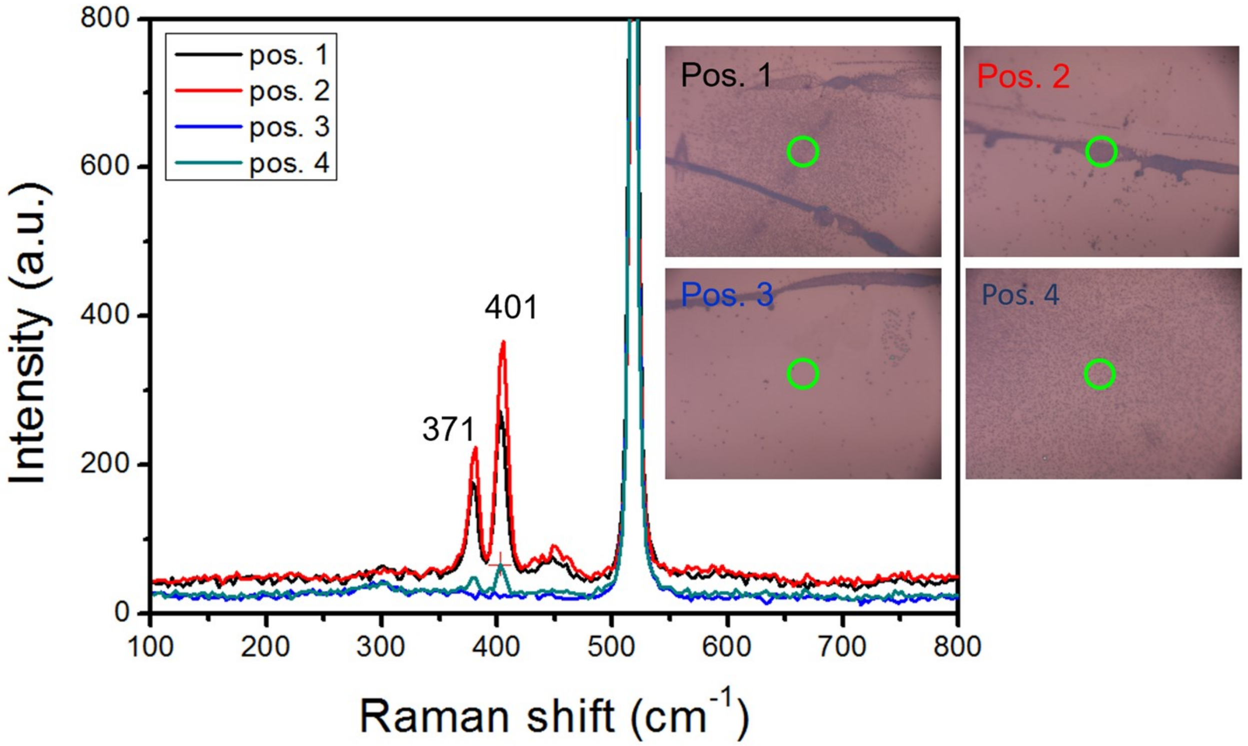

Figure S1. Raman spectra of transferred MoS_2 on SiO_2/Si substrate

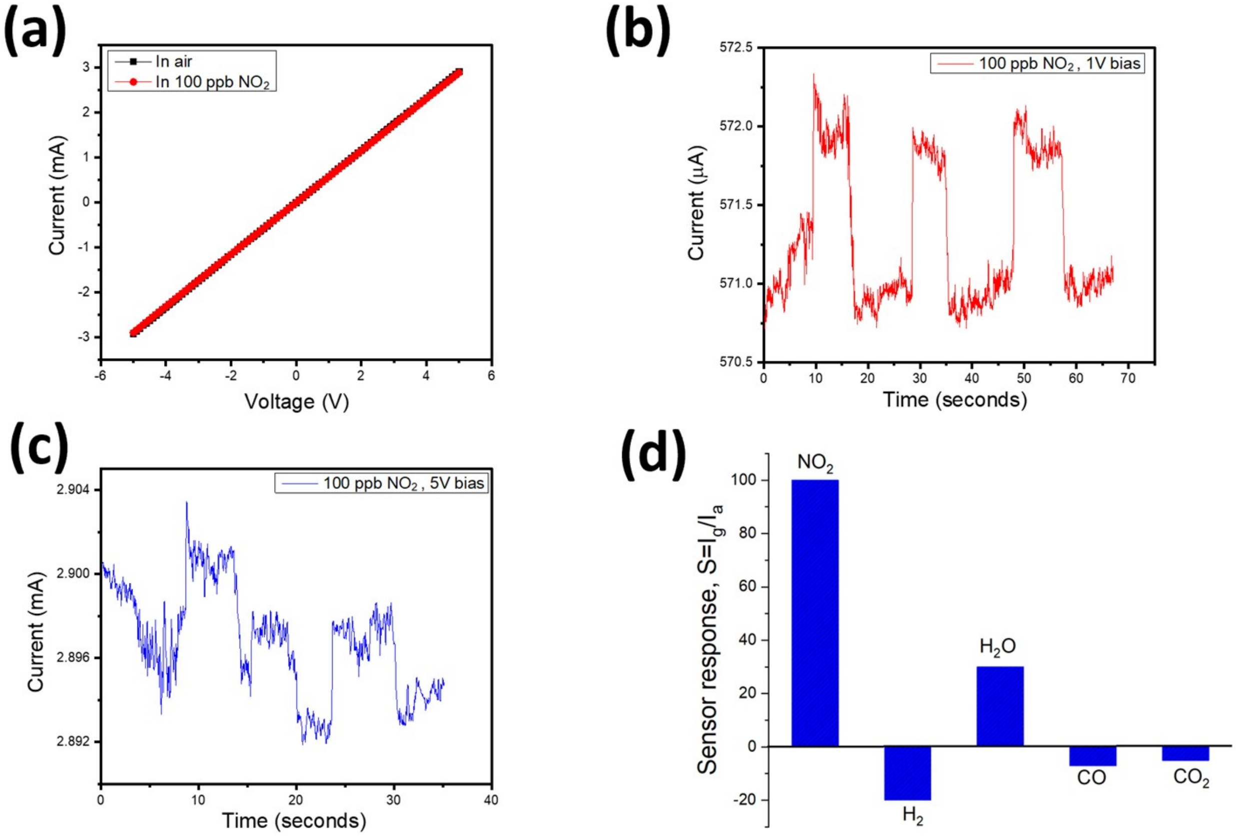

Figure S2. (a,b,c) The response of sensor with similar structure fabricated on pure graphene (without MoS_2) for comparison purpose, (d) the selectivity of fabricated sensor of Au-MoS_2-Au (measured at 1000 ppb).
